# Supplementary material for: Cassini: streamlined and scalable method for in situ profiling of RNA and protein
Source: Nat Commun. 2025 Oct 1;16:8747. doi: 10.1038/s41467-025-63798-0 (PMC12488881; doi:10.1038/s41467-025-63798-0)
Supplement: Supplementary file 7 — Description of Additional Supplementary Files [file 41467_2025_63798_MOESM7_ESM.pdf]

Supplementary Data 1: Price detail for 30 genes + 2 conjugated antibodies Cassini full run

Supplementary Data 2: Padlock probes for RNA detection

Supplementary Data 3: Fluorescent probes for amplicon detection

Supplementary Data 4: Other oligos
